# Impacts Over Time of Neighborhood-Scale Interventions to Control Ticks and Tick-Borne Disease Incidence

**DOI:** 10.1089/vbz.2022.0094

**Published:** 2023-03-06

**Authors:** Richard S. Ostfeld, Stacy Mowry, William Bremer, Shannon Duerr, Andrew S. Evans, Ilya R. Fischhoff, Alison F. Hinckley, Sarah A. Hook, Fiona Keating, Jennifer Pendleton, Ashley Pfister, Marissa Teator, Felicia Keesing

**Affiliations:** ^1^ Cary Institute of Ecosystem Studies, Millbrook, New York, USA.; ^2^ Department of Behavioral and Community Health, Dutchess County, Poughkeepsie, New York, USA.; ^3^ Centers for Disease Control and Prevention, Fort Collins, Colorado, USA.; ^4^ Bard College, Annandale, New York, USA.

**Keywords:** Lyme disease, blacklegged tick, *Ixodes scapularis*, integrated tick management, disease prevention

## Abstract

**Background:**

Controlling populations of ticks with biological or chemical acaricides is often advocated as a means of reducing human exposure to tick-borne diseases. Reducing tick abundance is expected to decrease immediate risk of tick encounters and disrupt pathogen transmission cycles, potentially reducing future exposure risk.

**Materials and Methods:**

We designed a placebo-controlled, randomized multiyear study to assess whether two methods of controlling ticks—tick control system (TCS) bait boxes and Met52 spray—reduced tick abundance, tick encounters with people and outdoor pets, and reported cases of tick-borne diseases. The study was conducted in 24 residential neighborhoods in a Lyme disease endemic zone in New York State. We tested the hypotheses that TCS bait boxes and Met52, alone or together, would be associated with increasing reductions in tick abundance, tick encounters, and cases of tick-borne disease over the 4–5 years of the study.

**Results:**

In neighborhoods with active TCS bait boxes, populations of blacklegged ticks (*Ixodes scapularis*) were not reduced over time in any of the three habitat types tested (forest, lawn, shrub/garden). There was no significant effect of Met52 on tick abundance overall, and there was no evidence for a compounding effect over time. Similarly, we observed no significant effect of either of the two tick control methods, used singly or together, on tick encounters or on reported cases of tick-borne diseases in humans overall, and there was no compounding effect over time. Thus, our hypothesis that effects of interventions would accumulate through time was not supported.

**Conclusions:**

The apparent inability of the selected tick control methods to reduce risk and incidence of tick-borne diseases after years of use requires further consideration.

## Introduction

Rapid increases in incidence rates and geographic ranges (Petersen et al, 2019; Rosenberg et al, 2018) of tick-borne diseases have stimulated efforts to reduce human exposure. Along with vaccination (Heinz et al, 2013; Shen et al, 2011) and behavioral avoidance of vectors (Fischhoff et al, 2019), reducing the size of vector populations is considered a promising approach (Eisen and Stafford, 2021). Vector control is increasingly seen as a key component of the prevention of Lyme disease, which is the most frequently reported vector-borne disease in North America (Schwartz et al, 2017). The efficacy of both chemical and biological agents lethal to ticks has been assessed most often using relatively small-scale, short-term field studies (Eisen and Stafford, 2021). These studies suggest that intensive deployment of entomopathogenic agents can reduce the size of tick populations by upward of 50%.

Recently, we described a large-scale field study that tested the effects of two tick control interventions on tick numbers, human–tick encounters, and incidence of tick-borne disease in humans and outdoor pets (Keesing et al, 2022). The study was conducted in residential neighborhoods within an endemic area for Lyme disease (Keesing et al, 2022). In that study, we deployed tick control system (TCS) bait boxes, which use fipronil to kill ticks on small mammal hosts, and the fungal acaricide Met52, which kills questing ticks, or both, for four consecutive years (2017–2020). Each treatment was paired with a placebo control. Analyzing the aggregated results of 3 years of deployments and responses (2017–2019), we found that both questing blacklegged ticks (*Ixodes scapularis*) and ticks attached to small mammals were reduced by ∼50% in neighborhoods with active TCS bait boxes, compared with their placebo controls, whereas Met52 had a more modest effect in reducing tick numbers (Keesing et al, 2022).

Despite this reduction in abundance of blacklegged ticks, we found no significant effects of either intervention or of both together on incidence of tick-borne diseases in people, compared with the effects of placebo controls (Keesing et al, 2022). These results suggested that neighborhood-scale deployment of these two tick-killing interventions is unlikely to comprise an effective means of protecting the public against tick-borne diseases, despite moderately strong control of tick population size. However, in that study (Keesing et al, 2022), we focused on the overall responses to treatment rather than the effects of the interventions through time, and we did not include the final year of data collection, which was delayed because of the COVID-19 pandemic.

In the present study, we test the hypothesis that the two tick-killing interventions give rise to cumulative changes in either the size of tick populations or the incidence of tick-borne disease in residents, or both. The expectation of a cumulative effect of the deployment of entomopathogenic agents arises for two reasons. If tick abundance is reduced from early deployments of entomopathogens, then the abundance of the next generation of ticks should be reduced via demographic inertia (*i.e.,* compounding effects of control on the subsequent generation). This inertia could be weakened by tick dispersal to and from areas outside the treatment zone. Because our sampling unit was a large neighborhood, thus potentially mitigating the effects of tick dispersal, we considered it plausible that there would be detectable demographic inertia. Such an effect would be detected from repeated sampling of tick abundance.

A second pathway arises from potential self-reinforcing feedbacks between tick numbers and the transmission of tick-borne pathogens. According to this pathway, any early reduction in tick abundance is likely to reduce the number of ticks infesting reservoir hosts, such as white-footed mice (*Peromyscus leucopus*) and eastern chipmunks (*Tamias striatus*) (Ostfeld et al, 2018), which in turn should reduce the subsequent abundance of infected ticks, capable of infecting hosts the next year. Lower infection prevalence in hosts, in turn, should result in reduced host-to-tick transmission and hence in reduced tick infection prevalence. Although we do not report on tick infection in the current study, we test whether there was a cumulative effect of tick reduction on self-reported cases of tick-borne diseases in people and outdoor pets.

## Materials and Methods

### Design of interventions

The study took place in Dutchess County, New York, which has experienced high incidence rates of Lyme disease and other tick-borne diseases since the 1990s (Eisen et al, 2016). We selected two tick control interventions that had been demonstrated in small-scale studies to be effective in reducing population size of blacklegged ticks and that were considered safe for people, pets, and the environment (Dolan et al, 2004; Schulze et al, 2017; Schulze et al, 2007; Williams et al, 2018).

One intervention was the deployment of TCS bait boxes, which attract small mammals to a food source inside an enclosed device and apply the tick-killing chemical fipronil to these mammals. Fipronil is lethal to ticks but harmless to mammals (Dolan et al, 2004). The other intervention was the biopesticide Met52, which consists of spores of the F52 strain of the fungus *Metarhizium brunneum*. Met52 solution is mixed with water and sprayed on the ground and low-lying vegetation where ticks dwell. By killing ticks attached to small mammals, TCS bait boxes are expected to affect the abundance of host-seeking (questing) ticks the following year, whereas Met52 targets host-seeking ticks, with impacts expected within days to weeks after deployment.

As previously described in detail (Keesing et al, 2022), we selected 24 residential neighborhoods that had reported high incidence of tick-borne diseases in Dutchess County in recent prior years. Neighborhoods consisted of ∼100 adjacent 1- and 2-family residences at moderate to high density, including their respective yards. Overall average property size was 0.19 ha. After a year of exhaustive efforts to recruit eligible households in each neighborhood to participate in the study, we enrolled a mean of 34% of properties in each neighborhood (range 24–44%).

Each of the neighborhoods was randomly assigned to one of four treatment categories, with six neighborhoods assigned to each category: (1) active TCS bait boxes and active Met52; (2) active TCS bait boxes and placebo Met52; (3) placebo TCS bait boxes and active Met52; and (4) placebo TCS bait boxes and placebo Met52. All participating properties in each neighborhood received the same treatment category. Placebo TCS bait boxes were identical to active bait boxes except that they contained no fipronil. Placebo Met52 consisted of water only. Participating households agreed not to deploy broadcast acaricides independent of our study throughout its duration.

Both products were used according to label instructions. TCS bait boxes, covered with galvanized steel shrouds (active and placebo), were deployed twice annually, in spring and mid-summer, at an average rate of 5.9 boxes per property (38/ha), at least 10 meters apart, preferentially in sites frequented by small mammals. Active Met52 was sprayed by truck-mounted high-pressure sprayers (GNC Industries, Inc.) at a concentration of 2.22 L per 378.5 L of water. Placebo Met52 (water only) was sprayed using the same truck-mounted sprayers at the same rate of 4 L of spray per 93 m^2^ at a pressure of 1.2–1.4 MPa. Spraying of active and placebo Met52 occurred twice each year, immediately before (April—early May) and during (late May–late June) the peak activity period for nymphal blacklegged ticks in this region (Ostfeld et al, 2018). Further details are provided in the supplementary online appendix of Keesing et al (2022) (https://wwwnc.cdc.gov/eid/article/28/5/21-1146_article).

The study design was double-masked ( = “double-blind”), in that neither the members of participating households nor the team of researchers collecting data were aware of the treatment category of any of the neighborhoods. All data collection, entry, and compilation were conducted with the treatment categories remaining masked.

### Response variables

We were interested in the impacts of TCS bait boxes and Met52, compared with their placebo controls, on the abundance of questing blacklegged ticks, rates of tick infestation of small mammal hosts, encounters between study participants and ticks, and incidence of tick-borne disease in people and their pets (Keesing et al, 2022). For assessment of tick abundance, we sampled for ticks at 20 properties in each neighborhood selected at random out of the pool of participating properties. We were particularly interested in nymphal abundance because of the importance of nymphs in pathogen transmission. During the seasonal peak in host-seeking activity of nymphs (May–July), we used 1 × 1 meter white corduroy cloths to sample low vegetation and the ground, conducting up to ten 30-s intervals of flagging in each of three habitat types on each property—forest, lawn, and shrub/garden (modified from Rulison et al, 2013).

We randomized the order in which neighborhoods were visited for sampling, which was always >2 weeks after Met52 application. Flagging was restricted to the hours between 09:00 and 17:30 h. We analyzed the abundance of questing nymphal ticks in May–early July of 2017–2019 and 2021. The year 2017 was the first year that treatments were applied. Effects of Met52 were expected to be detectable in 2017, whereas impacts of TCS bait boxes were not expected to be detectable until 2018. We sampled ticks twice on each property during the activity period of each year's nymphs, and we estimated tick abundance on a property using the maximum of these two samples.

Levels of tick infestation of small mammals were estimated by conducting a mark-recapture study at 10 randomly chosen properties per neighborhood. Trapping was conducted in 2017–2019 from early August to mid-September, to coincide with the seasonal peak in larval tick activity (Keesing et al, 2022). Nine trapping stations with two Sherman traps per station (stations ≥5 meters apart) were established. Traps were baited with oats and sunflower seeds, set in late afternoon, and checked in early morning for three consecutive days. Captured small mammals were individually marked with uniquely numbered monel ear tags. We counted the number of immature ticks observed on white-footed mice and eastern chipmunks at the time of their first capture in a trapping session. All animals were released at the point of capture immediately after handling. The Institutional Animal Care and Use Committee of the Cary Institute of Ecosystem Studies reviewed and approved protocols involving the live-trapping and handling of vertebrate animals.

To determine the number of cases of tick-borne diseases experienced by participating people and their pets, and to estimate the number of encounters with ticks, we relied on electronic or telephone surveys distributed to each participating household every 2 weeks throughout the study. In each biweekly survey, we requested information from the primary contact person in each household as to whether they or any other full-time resident of their household (including outdoor pets) had encountered a tick or been diagnosed with a case of tick-borne disease in the prior 2 weeks. The Institutional Review Board of the Cary Institute of Ecosystem Studies reviewed and approved protocols (#131-2016) involving human subjects. All participants were fully informed of study procedures, their legal rights and responsibilities, the general scientific benefits expected from the research, and their right to voluntary termination without penalty or censure.

### Timing of the study and statistical analyses

Tick control interventions were deployed from 2017 through 2020. Despite the public health emergency curtailing many activities following the regional emergence of COVID-19 in 2020, we were able to maintain our treatment schedule and biweekly surveys throughout that year. However, the COVID-19 pandemic prevented us from sampling ticks during the 2020 season. We resumed our schedule of tick sampling in 2021, assessing the effects of the prior years of interventions on questing nymphal ticks in the year following cessation of treatments.

Based on the expected mode of action of active TCS bait boxes, this intervention would not be expected to have an effect on questing nymphal ticks until 2018, the year following the initiation of treatments. In contrast, based on the expected mode of action, the active Met52 treatment would be expected to have a possible effect on questing nymphal ticks in the first year of the treatments. However, both interventions could affect the burden of ticks on small mammals in the first year of treatments, based on their presumed modes of action.

We analyzed all of our response variables through time, with year as a factor, which would detect differences among years that arose from differences in the timing of the effects of interventions. As described above, data from 2017 might constitute a baseline for response variables in the TCS bait box neighborhoods, but not for response variables in the Met52 treatments. Therefore, to examine the potentially uneven effects of timing of the two interventions, we also analyzed the abundance of questing nymphal ticks and the numbers of cases and encounters by people and pets excluding the initial year of treatment (2017).

### Data analysis

Data were analyzed using R (version 4.0.1) (R Core Team, 2020). We used the packages *tidyr* (Wickham and Henry, 2020), *dplyr* (Wickham et al, 2020), and *forcats* (Wickham, 2020) to format and manipulate data, and the packages *ggplot2* (Wickham, 2016) and *cowplot* (Wilke, 2017) for graphing. To tidy statistical data, we used the *broom.mixed* (Bolker and Robinson, 2020) package.

We estimated tick densities as the mean number of questing nymphal ticks per flagging interval, evaluating each habitat type (forest, lawn, shrub/garden) separately. We analyzed data at the neighborhood level, calculating the average maximum number of questing nymphal ticks per flagging interval, by taking the mean of the values from each sampled property within a neighborhood. We used linear mixed-effects models built using package *nlme* (Pinheiro et al, 2020) to conduct our analyses, transforming the data when required to conform to assumptions of tests. To account for repeated measures, we treated neighborhood as a random effect, and we included year, treatment, and an interaction between the presence of active TCS boxes and active Met52 treatments as fixed effects. These data were analyzed with the inclusion of the first year of treatments (2017–2021) and without that year (2018–2021).

We also analyzed data from each of the individual properties we sampled, considering each habitat type (forest, lawn, shrub/garden) separately. For these property-level analyses, we only included data from properties at which we were able to conduct fieldwork in all 4 years of sampling—2017, 2018, 2019, and 2021. The number of properties for which we had data for all 4 sampling sessions ranged from 8 to 15 per neighborhood (mean 12.0 ± 0.43 standard error of the mean). Zero values for the number of questing ticks on a property were common, representing 43–82% of our property-level data.

To evaluate these data at the property level, we used generalized linear mixed models with a binomial distribution and a logit link function to fit a logistic model to the zero values versus the non-zero values. Our models included fixed effects of treatment, year, and an interaction between the presence of bait boxes and Met52, with a random effect of property nested within neighborhood. These analyses were conducted with the *glmmTMB* function in package *glmmTMB* (Brooks et al, 2017). We tested whether our data met the assumptions of our models using the *DHARMa* package (Hartig, 2020). We did not attempt to fit a generalized linear model to the non-zero values because the non-zero values did not conform to assumptions of tests. These data were analyzed with the inclusion of the first year of treatments (2017–2021) and without that year (2018–2021).

For data on the tick burdens on white-footed mice and eastern chipmunks, we calculated the weighted mean of the number of larval and nymphal ticks on each species, using the number of unique individuals (mice or chipmunks) on each property as the weighting factor. Properties for which there were no captures for a particular species were excluded from calculations of that neighborhood's mean tick burdens for that species for that year. We treated neighborhood as a random effect in our statistical models, with year, treatment, and an interaction between the presence of active TCS boxes and active Met52 treatments as fixed effects. The weighted means of ticks on mice were analyzed using linear mixed-effects models with package *nlme* (Pinheiro et al, 2020), using transformations of the data to conform to assumptions of tests.

The weighted means of ticks on chipmunks did not conform to the assumptions of linear models, so we first used a generalized linear mixed model with a binomial distribution and a logit link function to fit a logistic model to the zero values versus the non-zero values. We then used a generalized linear mixed model with a gamma distribution for the non-zero values, with neighborhood as a random effect, and year and an interaction between the use of active TCS boxes and/or Met52 spray as fixed effects. These analyses were conducted with the *glmmTMB* function in package *glmmTMB* (Brooks et al, 2017). We tested whether our data met the assumptions of the models using the *DHARMa* package (Hartig, 2020). These data were analyzed with the inclusion of the first year of treatments (2017–2021).

Our data for encounters with ticks and cases of tick-borne diseases for both humans and pets were gathered from a total of 78 biweekly surveys, which we administered to participants from May 2017 through December 2020 (see table S2 in Keesing et al, 2022). For all case and encounter data, we included in our analyses estimates of the number of participating people or pets in a neighborhood over a particular year. The number of people and pets was determined by surveys conducted at the start or end of each field season. If a participating household did not respond to a specific survey, we assumed the number of people and pets remained the same as in the prior survey.

We used these data to calculate the number of human and pet participants in a neighborhood during each biweekly survey period and then averaged these biweekly estimates to establish the annual number of participants. If participants informed us of changes to the number of residents or pets in their household during a season, these changes were incorporated at the time of the relevant biweekly survey. Members of households that withdrew from the study were not included in analyses of cases for seasons in which they were enrolled for fewer than half of the biweekly surveys.

We calculated the number of reported encounters by people or pets for all participating households in a neighborhood based on participant responses to our biweekly surveys. A specific tick encounter reported by a participant was calculated as a binary value (yes/no), regardless of the number of ticks the participant reported having encountered. To be included in a particular year's data on encounters or cases, a household needed to have responded to at least one of that year's biweekly surveys.

We analyzed the number of human or pet cases using self-reported cases of tick-borne diseases indicated by participants during biweekly surveys. For these self-reports, participants were asked to report household cases of tick-borne diseases diagnosed by a health care provider.

For reported tick encounters and cases of tick-borne diseases for people and pets, we calculated the number of reports of each type for each neighborhood for each of the 4 years of treatments (2017–2020). Because our data relied on responses to our biweekly surveys, our estimates of the number of participants in a neighborhood included only participants (or pets) from households that submitted at least one biweekly survey in a given year. We used generalized linear models with treatment, year, and an interaction between the presence of active TCS boxes and active Met52 as fixed effects, with an offset for the mean number of people (or pets) in a neighborhood.

We used the *glmmTMB* function in package *glmmTMB* (Brooks et al, 2017) to fit models with a negative binomial distribution. We evaluated the residuals of the models (*e.g.,* for overdispersion) using the *DHARMa* package (Hartig, 2020). When necessary to account for a large number of zero values in a particular response variable, we included a term for zero-inflation. These data were analyzed with the inclusion of the first year of treatments (2017–2020) and without that year (2018–2020).

## Results

### Tick abundance: questing nymphs

**FIG. 1.**
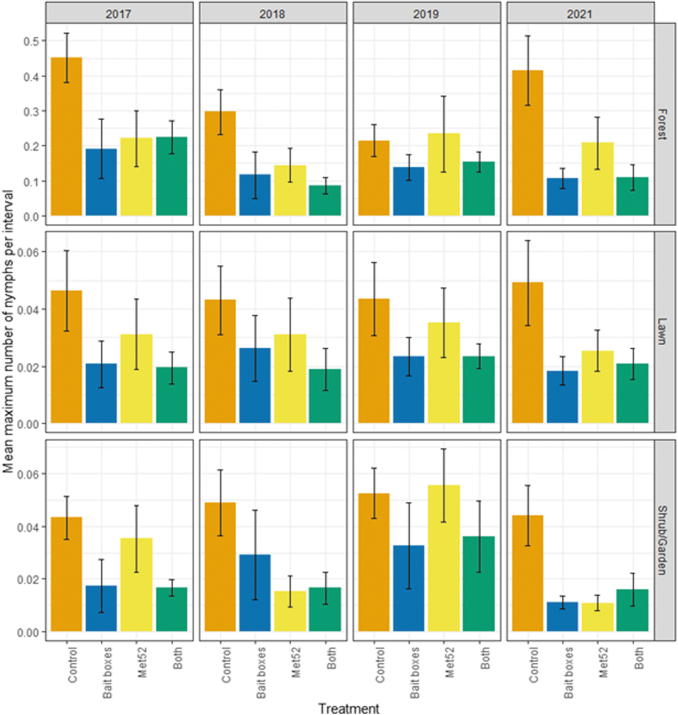
Abundance of host-seeking (questing) nymphal blacklegged ticks, represented by the weighted mean number of nymphal ticks per flagging interval as a function of experimental treatment and year. Means represent the average values of the six neighborhoods assigned to each treatment group. Error bars are standard errors. On the *x*-axis, Control indicates neighborhoods that had placebo controls for both TCS bait boxes and Met52, Bait boxes indicate neighborhoods that had active bait boxes but placebo Met52, Met52 indicates neighborhoods that had active Met52 but placebo bait boxes, and Both indicate neighborhoods that had active TCS bait boxes and active Met52. Note that the *y*-axis scales differently for each habitat type. TCS, tick control system.

Of the 38,551 questing ticks collected, ∼99% were identified as *I. scapularis*. Other species detected (*N* = 578 ticks altogether) included *Dermacentor variabilis*, *Haemaphysalis longicornis*, *Haemaphysalis leporispalustris*, and *Ixodes marxi*. Blacklegged tick abundance was roughly one order of magnitude higher in forest than in lawn or shrub/garden habitats (Fig. 1). In forest habitat, the abundance of questing nymphs was significantly lower in neighborhoods with active bait boxes compared with placebo controls (*p* = 0.02; Table 1), whereas tick abundance in neighborhoods with active Met52 and those with both active treatments were not significantly reduced, compared with placebo controls (Table 1).

**Table 1.** Results of the Statistical Analyses of Abundance of Questing Blacklegged Ticks Across the Neighborhoods (*N* = 6) Assigned to Each Treatment

Effect	Group	Term	Estimate	SE	df	t	*p*
Forest habitat 2017–2021							
Fixed		(Intercept)	21.08	14.62	71	1.44	0.15
Fixed		Year	−0.01	0.01	71	−1.40	0.16
Fixed		Active Bait Box	−0.23	0.09	20	−2.64	0.02
Fixed		Active Met52	−0.16	0.09	20	−1.87	0.08
Fixed		Active Bait Box and Met52	0.18	0.12	20	1.45	0.16
Random	Neighborhood	sd_(Intercept)	0.14				
Random	Residual	sd_Observation	0.10				
Lawn habitat 2017–2021							
Fixed		(Intercept)	−1.98	6.84	71	−0.29	0.77
Fixed		Year	0.00	0.00	71	0.32	0.75
Fixed		Active Bait Box	−0.07	0.03	20	−1.92	0.07
Fixed		Active Met52	−0.04	0.03	20	−1.28	0.22
Fixed		Active Bait Box and Met52	0.04	0.05	20	0.75	0.46
Random	Neighborhood	sd_(Intercept)	0.05				
Random	Residual	sd_Observation	0.05				
Shrub habitat 2017–2021							
Fixed		(Intercept)	6.54	9.59	71	0.68	0.50
Fixed		Year	0.00	0.00	71	−0.66	0.51
Fixed		Active Bait Box	−0.09	0.03	20	−2.80	0.01
Fixed		Active Met52	−0.06	0.03	20	−2.03	0.06
Fixed		Active Bait Box and Met52	0.07	0.04	20	1.68	0.11
Random	Neighborhood	sd_(Intercept)	0.04				
Random	Residual	sd_Observation	0.07				

Data from 2017 to 2021 (excluding 2020) are included. Analyses were conducted separately for each habitat type sampled.

SE, standard error.

For lawn and shrub/garden habitats, results were qualitatively similar, with tick abundance generally lower in neighborhoods with active bait boxes (not significant in lawn) but with more modest and nonsignificant effects of active Met52 and both treatments combined (Table 1). The effect of Year was not significant for any habitat type (*p* ≥ 0.16), indicating that the hypothesized reductions over time were not observed (Table 1).

The abundance of questing nymphs in neighborhoods with active bait boxes was lower in all habitats in 2017 (Fig. 1), the year of initial deployment of bait boxes, compared with placebo controls. Results were qualitatively similar when we analyzed the results for 2018–2021 only, excluding 2017 as a baseline year. For forest, the effect of active bait boxes was significant (*p* = 0.02), but that of active Met52 and of both active treatments were not significant (Table 2). For lawn, the effect of active bait boxes was weaker and nonsignificant (*p* = 0.08), and for shrub/garden, the effects of both active bait boxes (*p* = 0.02) and active Met52 (*p* = 0.04) were significant (Table 2). In no case was the effect of Year significant.

**Table 2.** Results of the Statistical Analyses of Abundance of Questing Blacklegged Ticks Across the Neighborhoods (*N* = 6) Assigned to Each Treatment

Effect	Group	Term	Estimate	SE	df	t	*p*
Forest habitat 2018–2021							
Fixed		(Intercept)	−37.97	21.90	47	−1.73	0.09
Fixed		Year	0.02	0.01	47	1.71	0.09
Fixed		Active Bait Box	−0.27	0.11	20	−2.57	0.02
Fixed		Active Met52	−0.18	0.11	20	−1.67	0.11
Fixed		Active Bait Box and Met52	0.18	0.15	20	1.22	0.24
Random	Neighborhood	sd_(Intercept)	0.17				
Random	Residual	sd_Observation	0.11				
Lawn habitat 2018–2021							
Fixed		(Intercept)	0.03	8.96	47	0.00	1.00
Fixed		Year	0.00	0.00	47	0.02	0.98
Fixed		Active Bait Box	−0.06	0.03	20	−1.87	0.08
Fixed		Active Met52	−0.04	0.03	20	−1.21	0.24
Fixed		Active Bait Box and Met52	0.03	0.05	20	0.70	0.49
Random	Neighborhood	sd_(Intercept)	0.05				
Random	Residual	sd_Observation	0.05				
Shrub habitat 2018–2021							
Fixed		(Intercept)	14.96	14.37	47	1.04	0.30
Fixed		Year	−0.01	0.01	47	−1.03	0.31
Fixed		Active Bait Box	−0.08	0.03	20	−2.63	0.02
Fixed		Active Met52	−0.07	0.03	20	−2.20	0.04
Fixed		Active Bait Box and Met52	0.08	0.05	20	1.73	0.10
Random	Neighborhood	sd_(Intercept)	0.03				
Random	Residual	sd_Observation	0.08				

Data from 2018 to 2021 (excluding 2017 and 2020) are analyzed. Analyses are conducted separately for each habitat type sampled.

**FIG. 2.**
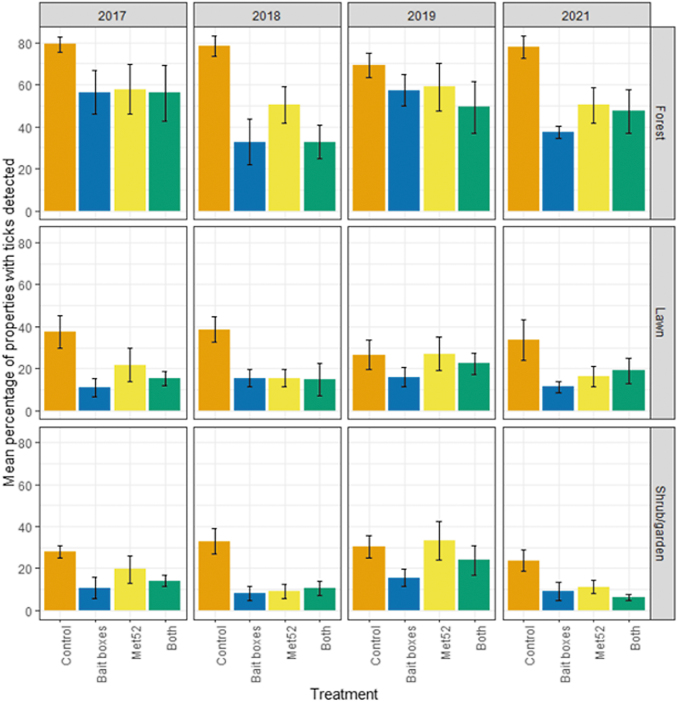
Abundance of host-seeking (questing) nymphal blacklegged ticks, indicated by the mean percentage of properties with nymphal ticks observed as a function of experimental treatment and year. Error bars are standard errors. On the *x*-axis, Control indicates neighborhoods that had placebo controls for both TCS bait boxes and Met52, Bait boxes indicate neighborhoods that had active bait boxes but placebo Met52, Met52 indicates neighborhoods that had active Met52 but placebo bait boxes, and Both indicate neighborhoods that had active TCS bait boxes and active Met52.

Our parallel analyses of questing nymphs at the level of individual properties within neighborhoods allowed us to ask whether the treatments were associated with differences in the percentage of properties that had detectable tick populations, versus those with no observed ticks. Through this analysis, we observed a reduced percentage of properties with detectable numbers of ticks in neighborhoods receiving active bait boxes and those receiving active Met52 treatments for forest, lawn, and shrub/garden (Fig. 2, Table 3, and Supplementary Table S1). Again, the effect of Year was not significant for any habitat type. Restricting analyses to sampling years after 2017 did not qualitatively affect results (Table 3).

**Table 3.** Results of the Statistical Analyses of the Percentage of Properties in Which Nymphal Blacklegged Ticks Were Observed, Across the Neighborhoods (*N* = 6) Assigned to Each Treatment

Effect	Group	Term	Estimate	SE	z	*p*
Forest habitat 2017–2021						
Fixed		(Intercept)	1.83	0.44	4.17	0.00
Fixed		Year	−0.12	0.07	−1.81	0.07
Fixed		Active Bait Box	−1.73	0.56	−3.08	0.00
Fixed		Active Met52	−1.25	0.56	−2.24	0.03
Fixed		Active Bait Box and Met52	1.23	0.79	1.56	0.12
Random	Property address	sd_(Intercept)	1.13			
Random	Neighborhood	sd_(Intercept)	0.82			
Lawn habitat 2017–2021						
Fixed		(Intercept)	−0.73	0.34	−2.13	0.03
Fixed		Year	−0.03	0.07	−0.39	0.70
Fixed		Active Bait Box	−1.38	0.44	−3.13	0.00
Fixed		Active Met52	−0.91	0.43	−2.10	0.04
Fixed		Active Bait Box and Met52	1.23	0.63	1.97	0.05
Random	Property address	sd_(Intercept)	0.82			
Random	Neighborhood	sd_(Intercept)	0.62			
Shrub/garden habitat 2017–2021						
Fixed		(Intercept)	−0.91	0.27	−3.33	0.00
Fixed		Year	−0.03	0.07	−0.51	0.61
Fixed		Active Bait Box	−1.33	0.33	−4.01	0.00
Fixed		Active Met52	−0.68	0.31	−2.19	0.03
Fixed		Active Bait Box and Met52	0.95	0.47	2.03	0.04
Random	Property address	sd_(Intercept)	0.64			
Random	Neighborhood	sd_(Intercept)	0.37			

Data from 2017 to 2021 (excluding 2020) are analyzed. Analyses are conducted separately for each habitat type sampled.

### Tick abundance: ticks on rodent hosts

**FIG. 3.**
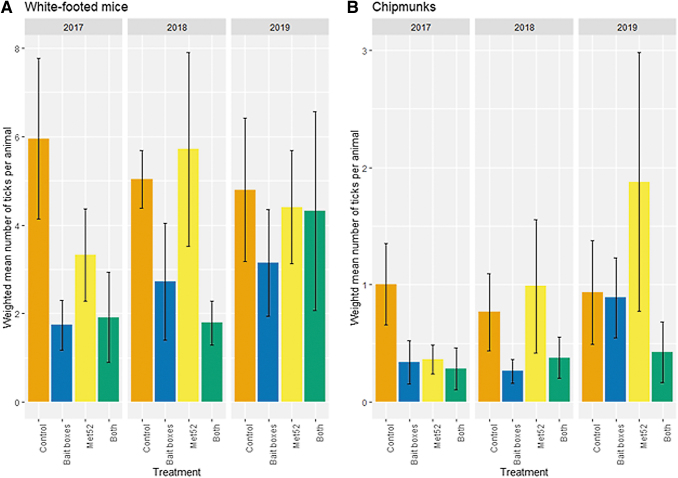
Abundance of ticks on small mammals, represented by the weighted average number of immature ticks per individual white-footed mouse **(A)** and eastern chipmunk **(B)**, as a function of experimental treatment and year. Averages represent the weighted mean values of the six neighborhoods assigned to each treatment group. Error bars are standard errors. On the *x*-axis, Control indicates neighborhoods that had placebo controls for both TCS bait boxes and Met52, Bait boxes indicate neighborhoods that had active bait boxes but placebo Met52, Met52 indicates neighborhoods that had active Met52 but placebo bait boxes, and Both indicate neighborhoods that had active TCS bait boxes and active Met52. The scale of the *y*-axis differs between panels.

Neighborhoods with active bait boxes were associated with reduced numbers of immature ticks on white-footed mice, compared with placebo controls (*p* = 0.03; Fig. 3 and Table 4). Neighborhoods with active Met52 and with both active treatments did not significantly differ from controls, and the effect of Year was not significant (Table 4). For ticks on chipmunks, the large number of tick-free individual hosts necessitated the use of a hurdle-modeling approach to analyze factors affecting first the frequency of tick-free hosts, and secondarily, the numbers of ticks on hosts with ≥1 tick. For both of these analyses, there were no significant effects of active bait boxes, active Met52, both active treatments together, or Year (Table 4).

**Table 4.** Results of Statistical Analyses of the Weighted Mean of Ticks on Mice 2017–2019 (A) and 2018–2019 (B), and of Ticks on Chipmunks 2017–2019 (C) and 2018–2019 (D)

Effect	Group	Term	Estimate	SE	df	t	*p*
A. Weighted mean of ticks on mice 2017–2019							
Fixed		(Intercept)	−340.05	222.00	47	−1.53	0.13
Fixed		Year	0.17	0.11	47	1.54	0.13
Fixed		Active Bait Box	−0.80	0.34	20	−2.37	0.03
Fixed		Active Met52	−0.26	0.34	20	−0.77	0.45
Fixed		Active Bait Box and Met52	0.26	0.48	20	0.55	0.59
Random	Neighborhood	sd_(Intercept)	0.39				
Random	Residual	sd_Observation	0.76				
B. Weighted mean of ticks on mice 2018–2019							
Fixed		(Intercept)	−264.82	476.63	23	−0.56	0.58
Fixed		Year	0.13	0.24	23	0.56	0.58
Fixed		Active Bait Box	−0.62	0.36	20	−1.71	0.10
Fixed		Active Met52	−0.05	0.36	20	−0.14	0.89
Fixed		Active Bait Box and Met52	0.08	0.51	20	0.16	0.87
Random	Neighborhood	sd_(Intercept)	0.25				
Random	Residual	sd_Observation	0.82				

Effect	Group	Term	Estimate	SE	z	*p*	
C. Weighted mean of ticks on chipmunks							
For chipmunk 2017–2019, zero values							
Fixed		(Intercept)	23.06	21235.89	0.00	1.00	
Fixed		2018	2.77	1.48	1.87	0.06	
Fixed		2019	2.77	1.48	1.87	0.06	
Fixed		Active Bait Box	−23.46	21235.89	0.00	1.00	
Fixed		Active Met52	−19.92	21235.89	0.00	1.00	
Fixed		Active Bait Box and Met52	19.22	21235.89	0.00	1.00	
Random	Neighborhood	sd_(Intercept)	2.19				

Effect	Component	Group	Term	Estimate	SE	z	*p*
For chipmunk 2017–2019, non-zero values							
Fixed	cond		(Intercept)	−0.23	0.33	−0.70	0.49
Fixed	cond		2018	−0.28	0.29	−0.97	0.33
Fixed	cond		2019	0.25	0.30	0.83	0.41
Fixed	cond		Active Bait Box	−0.12	0.44	−0.27	0.79
Fixed	cond		Active Met52	0.01	0.41	0.02	0.98
Fixed	cond		Active Bait Box and Met52	−0.18	0.63	−0.28	0.78
Random	cond	Neighborhood	sd_(Intercept)	0.50			

Effect	Group	Term	Estimate	SE	z	*p*	
D. Weighted mean of ticks on chipmunks							
For chipmunk 2018–2019, zero values							
Fixed		(Intercept)	18.47	415.08	0.04	0.96	
Fixed		2019	0.00	1.91	0.00	1.00	
Fixed		Active Bait Box	−11.28	415.08	−0.03	0.98	
Fixed		Active Met52	1.21	865.27	0.00	1.00	
Fixed		Active Bait Box and Met52	−1.21	865.28	0.00	1.00	
Random	Neighborhood	sd_(Intercept)	9.97				

Effect	Group	Term	Estimate	SE	z	*p*	
For chipmunk 2018–2019, non-zero values							
Fixed		(Intercept)	−0.60	0.37	−1.63	0.10	
Fixed		2019	0.48	0.25	1.89	0.06	
Fixed		Active Bait Box	−0.03	0.53	−0.05	0.96	
Fixed		Active Met52	0.21	0.50	0.43	0.67	
Fixed		Active Bait Box and Met52	−0.60	0.75	−0.79	0.43	
Random	Neighborhood	sd_(Intercept)	0.65				

Parts C and D reflect a hurdle-modeling approach to separately treat hosts with zero and non-zero values and are thus divided into these two components.

### Encounters with ticks and cases of tick-borne diseases: humans

**FIG. 4.**
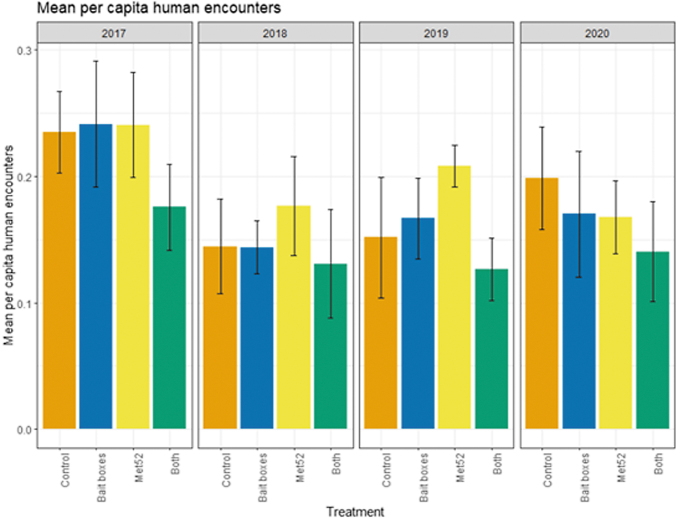
Mean per capita human encounters with ticks, self-reported, as a function of experimental treatment and year. Error bars are standard errors. On the *x*-axis, Control indicates neighborhoods that had placebo controls for both TCS bait boxes and Met52, Bait boxes indicate neighborhoods that had active bait boxes but placebo Met52, Met52 indicates neighborhoods that had active Met52 but placebo bait boxes, and Both indicate neighborhoods that had active TCS bait boxes and active Met52.

**FIG. 5.**
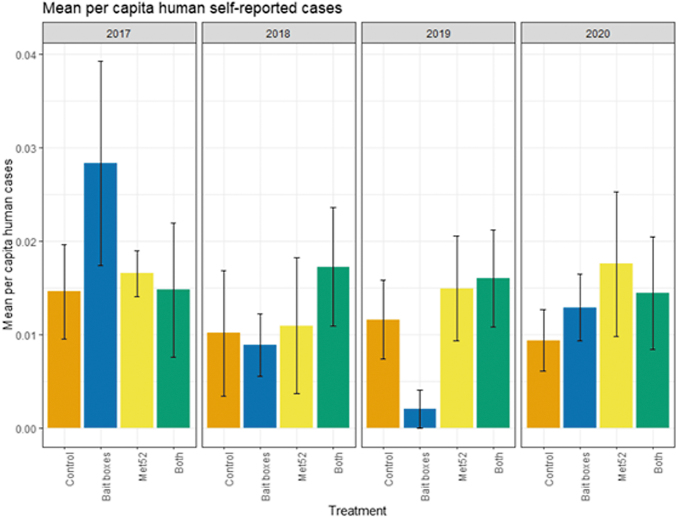
Mean per capita cases of tick-borne diseases in humans, self-reported, as a function of experimental treatment and year. Error bars are standard errors. On the *x*-axis, Control indicates neighborhoods that had placebo controls for both TCS bait boxes and Met52, Bait boxes indicate neighborhoods that had active bait boxes but placebo Met52, Met52 indicates neighborhoods that had active Met52 but placebo bait boxes, and Both indicate neighborhoods that had active TCS bait boxes and active Met52.

Based on data from 2017 through 2020, we reported previously that neither of the active treatments nor both of them combined were associated with reduced encounters with ticks or self-reported cases of tick-borne illness (Keesing et al 2022). In the current analysis, we found that the cumulative results previously reported were consistent across years. Neighborhoods with active bait boxes, active Met52, and both active treatments were not associated with significantly lower human encounters with ticks or cases of tick-borne disease, nor was the effect of Year significant (Figs. 4 and 5 and Tables 5 and 6). In the analysis of data from 2018 to 2020 (removing 2017), the results did not change qualitatively (Tables 5 and 6).

**Table 5.** Statistical Results of the Analysis of Annual Human Self-Reported Encounters with Ticks for 2017–2020 and for 2018–2020 with an Offset for Number of People in Neighborhood

Term	Estimate	SE	z	*p*
2017–2020				
(Intercept)	169.37	96.51	1.76	0.08
Year	−0.08	0.05	−1.77	0.08
Active Bait Box	0.01	0.14	0.04	0.97
Active Met52	0.16	0.14	1.17	0.24
Active Bait Box and Met52	−0.39	0.21	−1.86	0.06
2018–2020				
(Intercept)	−97.92	149.81	−0.65	0.51
Year	0.05	0.07	0.64	0.52
Active Bait Box	0.00	0.17	0.02	0.99
Active Met52	0.20	0.16	1.23	0.22
Active Bait Box and Met52	−0.42	0.25	−1.71	0.09

**Table 6.** Statistical Results of the Analysis of Annual Human Self-Reported Diagnoses for 2017–2020 and for 2018–2020 with an Offset for Number of People in Neighborhood

Term	Estimate	SE	z	*p*
2017–2020				
(Intercept)	189.96	178.97	1.06	0.29
Year	−0.10	0.09	−1.09	0.28
Active Bait Box	0.07	0.29	0.22	0.82
Active Met52	0.31	0.27	1.13	0.26
Active Bait Box and Met52	−0.03	0.39	−0.07	0.95
2018–2020				
(Intercept)	−123.03	297.25	−0.41	0.68
Year	0.06	0.15	0.40	0.69
Active Bait Box	−0.25	0.38	−0.67	0.50
Active Met52	0.35	0.33	1.07	0.28
Active Bait Box and Met52	0.36	0.49	0.74	0.46

### Encounters with ticks and cases of tick-borne diseases: outdoor pets

**FIG. 6.**
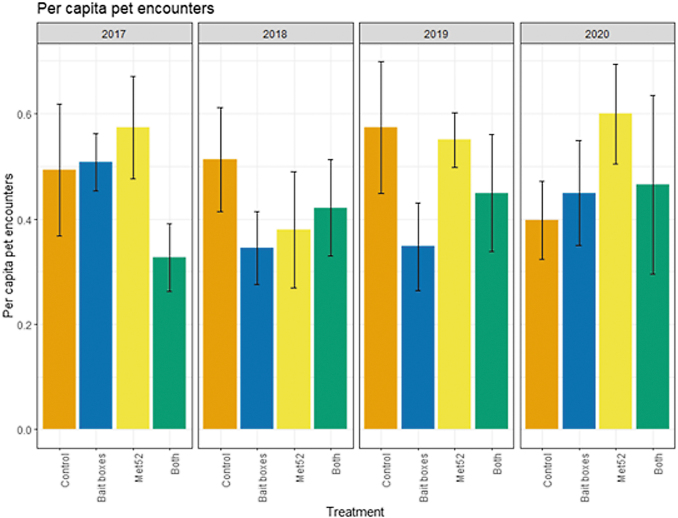
Mean per capita outdoor pet encounters with ticks as a function of experimental treatment and year. Error bars are standard errors. On the *x*-axis, Control indicates neighborhoods that had placebo controls for both TCS bait boxes and Met52, Bait boxes indicate neighborhoods that had active bait boxes but placebo Met52, Met52 indicates neighborhoods that had active Met52 but placebo bait boxes, and Both indicate neighborhoods that had active TCS bait boxes and active Met52.

**FIG. 7.**
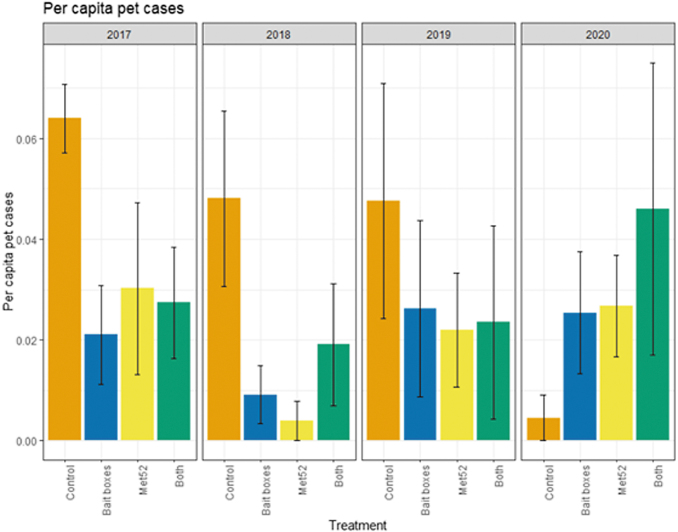
Mean per capita cases of tick-borne diseases in pets as a function of experimental treatment and year. Error bars are standard errors. On the *x*-axis, Control indicates neighborhoods that had placebo controls for both TCS bait boxes and Met52, Bait boxes indicate neighborhoods that had active bait boxes but placebo Met52, Met52 indicates neighborhoods that had active Met52 but placebo bait boxes, and Both indicate neighborhoods that had active TCS bait boxes and active Met52.

Based on the data from 2017 through 2020, we reported previously that neither of the active treatments nor both of them combined were associated with reduced cumulative pet encounters with ticks, but that cumulative cases of tick-borne disease in pets were significantly lower in both the active bait box and active Met52 neighborhoods. In the current analysis, we again found that the cumulative results previously detected were consistent in the analysis across years. For ticks found on pets, the effects of treatment and Year were not significant (Fig. 6 and Table 7). Neighborhoods with active bait boxes and active Met52 (but not both combined) were associated with significantly lower annual cases of tick-borne disease in pets (Fig. 7 and Table 8). However, when we conducted a second analysis on data only from 2018 to 2020, the effects of active bait boxes and active Met52 were no longer significant (Table 8). We did not detect a significant effect of Year in either analysis.

**Table 7.** Statistical Results of the Analysis of Annual Pet Encounters with Ticks for 2017–2020 and for 2018–2020 with an Offset for Number of Pets in Neighborhood

Term	Estimate	SE	z	*p*
2017–2020				
(Intercept)	−35.99	87.27	−0.41	0.68
Year	0.02	0.04	0.40	0.69
Active Bait Box	−0.11	0.14	−0.76	0.45
Active Met52	0.12	0.13	0.92	0.36
Active Bait Box and Met52	−0.24	0.19	−1.26	0.21
2018–2020				
(Intercept)	−130.13	140.05	−0.93	0.35
Year	0.06	0.07	0.92	0.36
Active Bait Box	−0.20	0.17	−1.20	0.23
Active Met52	0.09	0.15	0.60	0.55
Active Bait Box and Met52	−0.09	0.23	−0.40	0.69

**Table 8.** Statistical Results of the Analysis of Annual Cases of Tick-Borne Disease in Outdoor Pets for 2017–2020 and for 2018–2020 with an Offset for Number of Pets in Neighborhood

Term	Estimate	SE	z	*p*
2017–2020				
(Intercept)	172.43	209.13	0.82	0.41
Year	−0.09	0.10	−0.84	0.40
Active Bait Box	−0.67	0.32	−2.10	0.04
Active Met52	−0.70	0.31	−2.25	0.02
Active Bait Box and Met52	0.76	0.47	1.61	0.11
2018–2020				
(Intercept)	−208.60	346.39	−0.60	0.55
Year	0.10	0.17	0.59	0.55
Active Bait Box	−0.50	0.39	−1.29	0.20
Active Met52	−0.57	0.39	−1.48	0.14
Active Bait Box and Met52	0.56	0.57	0.98	0.33

## Discussion

This study was designed to evaluate whether two commercially available tick control interventions, deployed at the level of residential neighborhoods, reduced tick abundance, encounters with ticks, and cases of tick-borne disease in people and their outdoor pets. Previously, we reported (Keesing et al, 2022) that one of the interventions, TCS bait boxes, reduced abundance of questing nymphal blacklegged ticks by 53% compared with placebo controls. Abundance of ticks attached to small mammals also was reduced by ∼50%, whereas the other intervention, Met52, was not associated with significant reductions. We also reported that the interventions significantly reduced owner-reported cases of tick-borne diseases in outdoor pets, but that neither intervention (nor both combined) was associated with reductions in either human encounters with ticks or self-reported cases of tick-borne disease.

These prior results (Keesing et al, 2022) were based on aggregate or cumulative responses to experimental treatments observed over the period in which treatments were imposed. Here, we assessed whether the effects of the treatments changed through time and evaluated the impacts of the interventions on tick encounters and tick-borne disease cases for each year rather than as a cumulative total. We also included data on abundance of questing ticks for an additional year, 2021, which was the year following cessation of treatments.

Because none of our statistical tests of the effects of active interventions revealed a significant effect of Year, we conclude that impacts of TCS bait boxes and Met52 did not change through time. The addition of 2021 data on questing nymphal ticks did not change the results previously reported (Keesing et al, 2022) and reinforced the conclusion that active TCS bait boxes, but not active Met52, reduced the abundance of questing ticks compared with placebo controls. Surprisingly, tick abundance was not significantly reduced in neighborhoods with both active TCS bait boxes and active Met52.

We suspect that, rather than interfering with TCS bait boxes, Met52 increased variation in tick abundance independent of the effects of bait boxes. Assessment of tick encounters and cases of tick-borne disease from each year gave results consistent with those based on cumulative encounters and cases (Keesing et al, 2022). Although cases of owner-reported tick-borne diseases in outdoor pets were lower in neighborhoods with active TCS bait boxes and active Met52 when analyzed with 2017 data, neither tick encounters nor annual cases of tick-borne diseases in people were reduced by either treatment or by both combined.

Our observation that the abundance of questing nymphs was lower in neighborhoods with active TCS bait boxes in 2017, the first year of deployment, was unexpected. Because the fipronil in TCS bait boxes kills immature ticks on small mammal hosts, the effect of bait boxes on questing nymphal ticks is not expected in the first season of deployment. Instead, reduced larval burdens on small mammals in 1 year are expected to reduce questing nymphs the following year (Dolan et al, 2004). The lower abundance of questing nymphs in 2017 might have occurred because the 12 neighborhoods randomly selected to receive active TCS bait boxes (with or without active Met52) had smaller initial populations of ticks before the imposition of treatments, due to chance alone.

However, statistical analysis revealed no differences among neighborhoods assigned to the four treatment categories in key factors, such as the percentage of forest cover, fragmentation of the landscape, the percentage of lawn and garden cover, or the percentage of properties enrolled in the study (Keesing et al, 2022). Nevertheless, it remains possible that half of the neighborhoods in our study had significantly lower tick abundance in 2017, either as the result of chronically lower tick abundance in these neighborhoods or because they experienced a stochastic reduction in 2017. If the neighborhoods assigned active TCS bait boxes chronically experienced lower tick abundance independent of bait boxes, it is likely that TCS bait boxes were less effective than indicated by our statistical analyses.

If the lower abundance of ticks in those neighborhoods was the result of a stochastic decline in tick abundance specific to 2017, we would expect tick populations in those neighborhoods to rebound in 2018 unless bait boxes suppressed them after 2017. However, the removal of 2017 data did not affect our results or conclusions. In a randomized controlled trial in Connecticut, USA, Hinckley et al (2021) reported no effect of active (vs. placebo) TCS bait boxes on abundance of questing blacklegged ticks in either the year of deployment or the subsequent 2 years. In a result similar to the one reported here, Williams et al (2018) reported the strongest impacts of a combined TCS bait box and Met52 treatment in the initial year of deployment. Because their design did not include a treatment with only TCS bait boxes, whether the same-year effects were due entirely or in part to bait boxes could not be tested.

An alternative to pre-existing conditions among our neighborhoods is that there were same-year effects of bait boxes on questing nymphal ticks, a possibility that to our knowledge has not been thoroughly tested. If active TCS bait boxes reduced burdens of immature ticks on small mammals, this could potentially attract more questing ticks to these treated mammals. The attraction of questing ticks to hosts with lower body burdens, via density-dependent feeding, has received little attention but seems plausible (Kershenbaum et al, 2012). For example, Levin and Fish (1998) found that grooming intensity of white-footed mice in the laboratory decreased with decreasing infestation density by blacklegged ticks.

Thus, lower body burdens of mice exposed to active bait boxes could attract more questing immature ticks, thereby removing them from the questing pool, which is what is measured by flagging and dragging techniques. The potential for high availability of small mammals to reduce same-season abundance of questing immature ticks has been shown in field studies (Ostfeld et al, 2018). Nevertheless, until the potential for attraction of host-seeking ticks to small mammals is demonstrated to be sensitive to tick burdens, such a scenario remains speculative.

Any proportional reduction in tick abundance as a result of either or both of the interventions would establish a smaller baseline population the next year, which in turn could be further reduced. Thus, we expected that any reduction in the abundance of ticks early in the study would lead to compounding reductions in later years. We did not find evidence to support this expectation. Some prior studies testing the effects of TCS bait boxes and Met52 have spanned ≤2 years, preventing assessment of compounding effects (Little et al, 2020; Machtinger and Li, 2019). Longer term studies have produced mixed results.

Schulze et al (2017) and Schulze et al (2007) reported decreasing abundances of host-seeking blacklegged ticks in the second and third years after deployment of TCS bait boxes on individual properties, but these studies did not explicitly consider temporal changes. Williams et al (2018) deployed TCS bait boxes together with Met52 within individual residential properties in 2013 and estimated density of questing nymphal blacklegged ticks that same year and in the following 3 years. The highest measured effectiveness was in 2013, with effectiveness declining each subsequent year. In the final year, 2016, Met52 was unavailable, and the bait boxes alone showed no efficacy. We conclude that there is scant evidence for cumulative effects of TCS bait boxes or Met52 on tick abundance.

Studies using large-scale applications of chemical acaricides, rather than Met52 and TCS bait boxes, have found cumulative effects on blacklegged tick populations (Schulze et al, 2008). It is not known whether these cumulative effects arose because of the specific acaricide used or because of the scale of application. The occurrence of cumulative effects of acaricide on tick abundance could correlate positively with the scale of the application, with smaller areas more likely to experience immigration of ticks and their hosts from adjacent untreated areas. This hypothesis remains to be tested.

Our analyses of temporal variation in effects of the tick-killing treatments on human and pet encounters with ticks and cases of tick-borne disease in people confirmed prior analyses of the data aggregated over the 4 years of the study (Keesing et al, 2022); no significant effects of either treatment were observed in any year. As described above, it is possible that neighborhoods selected to receive the TCS bait box treatment maintained lower tick populations due to pre-existing conditions rather than the efficacy of the bait boxes. However, reduced numbers of questing ticks were not associated with fewer cases of tick-borne disease or tick encounters for residents of those neighborhoods, regardless of the causes of these reductions.

These results are consistent with those of Hinckley et al (2016). To the best of our knowledge, Hinckley et al (2021) and Hinckley et al (2016) are the only other randomized, placebo-controlled double-masked studies examining the effects of acaricidal treatments on tick abundance, tick encounters, and incidence of tick-borne disease. Although Hinckley et al (2016) found a 63% reduction in abundance of questing nymphal *I. scapularis* associated with bifenthrin treatment, they did not find concomitant effects on tick encounters or disease incidence. Much stronger reductions in tick abundance may be necessary to reduce tick encounters or cases of tick-borne disease in these communities. Hinckley et al (2021) found no significant effects of TCS bait boxes placed in yards and ecotones on the abundance of questing blacklegged ticks, human encounters with ticks, or cases of tick-borne disease.

Although we found no effect of either treatment, or both combined, on reported pet encounters with ticks, we did find that both active TCS bait boxes and active Met52 were associated with consistent reductions in cases of tick-borne disease across years in outdoor pets. However, when we removed the 2017 data, there were no significant effects of either treatment.

In conclusion, the lack of association between reduced abundance of host-seeking nymphal blacklegged ticks in residential areas receiving acaricidal treatments and the incidence of tick-borne diseases in those areas (this report) (Hinckley et al, 2016; Keesing et al, 2022) suggests that tick control for disease reduction needs new approaches. One key issue requiring attention is what causes the high levels of heterogeneity in tick abundance between sites and through time. Such heterogeneity reduces the ability of statistical tests to detect the effects of tick control treatments and may itself be an important cause of variable risk.

Studies of safe and effective methods for reducing tick populations will continue to be important, and their relevance to public health must become a greater focus of attention. The causes of the disjunction between tick control and disease reduction are poorly understood (Eisen and Stafford, 2021; Hinckley et al, 2016; Keesing et al, 2022). Future studies should address whether applications of acaricides at larger scales might reduce human exposure to tick-borne pathogens, whether nonlinear threshold effects govern the relationship between tick abundance and human exposure risk, and whether other locations besides residential neighborhoods are more effective targets for treatment.

## Supplementary Material

Supplemental data
