# Supplementary material for: Impacts Over Time of Neighborhood-Scale Interventions to Control Ticks and Tick-Borne Disease Incidence
Source: Vector Borne Zoonotic Dis. 2023 Mar 6;23(3):89–105. doi: 10.1089/vbz.2022.0094 (PMC9993163; doi:10.1089/vbz.2022.0094)
Supplement: Supplemental data [file Suppl_TableS1.docx]

**SUPPLEMENTAL TABLE 1.** QUESTING NYMPHS -- RESULTS FOR PROPERTY-LEVEL ANALYSIS 2018-2021

Questing nymphs in forest (2018-2021) – zero vs. non-zero properties

| effect | group | term | estimate | SE | z-value | p.value |
| --- | --- | --- | --- | --- | --- | --- |
| fixed |  | (Intercept) | 0.992675 | 0.429995 | 2.308576 | 0.020967 |
| fixed |  | Year | 0.133669 | 0.094698 | 1.411535 | 0.158087 |
| fixed |  | Active BaitBoxYes | -1.67015 | 0.544675 | -3.06632 | 0.002167 |
| fixed |  | Active Met52Yes | -1.15651 | 0.541248 | -2.13674 | 0.032619 |
| fixed |  | Active BaitBox and Met52 | 1.120002 | 0.763537 | 1.46686 | 0.142414 |
| random | Property.Address:Nh | sd__(Intercept) | 0.923514 |  |  |  |
| random | Nh | sd__(Intercept) | 0.808549 |  |  |  |

Questing nymphs in lawn (2018-2021) – zero vs non-zero properties

| effect | group | term | estimate | SE | z-value | p.value |
| --- | --- | --- | --- | --- | --- | --- |
| fixed |  | (Intercept) | -0.76321 | 0.372731 | -2.04762 | 0.040597 |
| fixed |  | Year | -0.06978 | 0.099918 | -0.69833 | 0.484974 |
| fixed |  | Active BaitBoxYes | -1.18439 | 0.463727 | -2.55406 | 0.010647 |
| fixed |  | Active Met52Yes | -0.84611 | 0.455202 | -1.85875 | 0.063062 |
| fixed |  | Active BaitBox and Met52 | 1.194537 | 0.656668 | 1.81909 | 0.068898 |
| random | Property.Address:Nh | sd__(Intercept) | 0.743803 |  |  |  |
| random | Nh | sd__(Intercept) | 0.649116 |  |  |  |

Questing nymphs in shrub/garden (2018-2021) – zero vs non-zero properties

| effect | group | term | estimate | SE | z-value | p.value |
| --- | --- | --- | --- | --- | --- | --- |
| fixed |  | (Intercept) | -0.95573 | 0.292449 | -3.26802 | 0.001083 |
| fixed |  | Year | -0.04347 | 0.10428 | -0.41687 | 0.676772 |
| fixed |  | Active BaitBoxYes | -1.23708 | 0.319586 | -3.87088 | 0.000108 |
| fixed |  | Active Met52Yes | -0.66841 | 0.294854 | -2.26693 | 0.023394 |
| fixed |  | Active BaitBox and Met52 | 0.872642 | 0.449346 | 1.942029 | 0.052134 |
| random | Property.Address:Nh | sd__(Intercept) | 0.729563 |  |  |  |
| random | Nh | sd__(Intercept) | 0.271353 |  |  |  |
